# Water quality, geochemistry and human health risk of groundwater in the Vyeboom region, Limpopo province, South Africa

**DOI:** 10.1038/s41598-023-46386-4

**Published:** 2023-11-04

**Authors:** Ntwanano Mutileni, Mulalo Mudau, Joshua Nosa Edokpayi

**Affiliations:** Water and Environmental Management Research Group, Faculty of Science, Engineering and Agriculture, University of Venda, Private Bag X5050, Thohoyandou, 0950 South Africa

**Keywords:** Microbiology, Environmental sciences, Chemistry

## Abstract

This study focuses on the evaluation of trace metals as well as microbial contamination of groundwater. Groundwater samples were collected from 17 boreholes. The microbial quality was tested using membrane filtration method. Higher levels of contamination for both *E. coli* and total coliform was recorded in the wet season. Majority of the boreholes had nitrate levels above the regulatory guideline value of the World health Organisation and the South African National Standards. The water type was established by Piper plot which showed the predominance of a magnesium bicarbonate water type, with alkaline earth metals dominating the alkali metals, as well as the weaker acids (bicarbonates) dominating the stronger ones (Sulphates and chlorides). Most of the trace metals detected were in compliance with the regulatory standard except for aluminium (0.41–0.88 mg/L). The hazard quotient and Hazard indice exceeded 1 mostly for children in both season which implies a possible non-carcinogenic health risk is associated with the continuous consumption of the water resource. The estimations of carcinogenic risk (CR_ing_) for Cr and Pb exceeded the carcinogenic indices of 10^−6^ and 10^−4^ which could pose adverse effects on human health for both children and adults. Therefore, it is recommended that measures should be implemented to reduce the risk.

## Introduction

The importance of water resources to human livelihood cannot be overemphasized. Unfortunately not everyone have sustainable access to clean and safe drinking water. The United Nation’s Sustainable development goal 6 has a vision to end such inconsistent access to portable water by the year 2023^1^. Despite the reported success, it is clear that sustainable access to clean and safe drinking water remains a wish for many especially those that live in the rural areas of developing countries. This is partly due to the absence of water infrastructure and erratic potable water supply^2,3^. To this end, most of the people often resort to alternative sources of drinking water with groundwater being the most prefeered option. Globally, WHO (World Health Organisation) and in South Africa, SANS (South African National Standard), set the guidelines for the parameters of portable water.

Groundwater is the source of drinking water in arid and semi-arid areas^4^ and its quality depends on the quality of the recharged water, rain, inland surface water and sub-surface geochemical processes^5^. These factors are in turn affected by suburbanisation, agricultural practices, industrial activities and climate change^6^. The temporal changes in the origin of the recharged water and hydrological human factors may also cause periodic changes in groundwater quality^5^. Thus, in order to ensure the quality of groundwater, previous research has recommended that scientific investigations should be used as a decision support system for policy adoption and groundwater designation in rural arid to semi-arid environments^7^.

Acoording to the International Association of Hydrogeologists, roughly one-third of the global population considers groundwater as being safe for human consumption^8^. There are also various misconceptions about groundwater that it is free from chemical and pathogenic contamination due to its aesthetic property. However, groundwater is exposed to heavy metal contamination which could be geogenic or anthropogenic in nature as well as microorganisms. Pollutants due to the use of fertilizers and the inadequate disposal of human and animal waste are other point sources of pollution in groundwater. Therefore aquifer geology and geochemical processes involved make groundwater pollution a complex subject^9,10^.

Rural areas in most developing countries are often characterized by animal husbandry, subsistence agriculture and inadequate solid waste management which also posses a potential threat to groundwater quality besides the factors that were highlted earlier. Most of the private and communual boreholes in rural areas are not routinely checked for water quality as users perceived they are safe for consumption. Cases of high fluoride, arsenic, nitrate and mercury levels have been reported in groundwater and there is a need to monitor their chemical and microbial composition. This study was carried out to assess the water quality and potential health risk associated with the levels of trace metals determined. The water type of the boreholes were also investigated.

The study area is Vyeboom region, located in Collins Chabane municipality of the Vhembe District, Limpopo Province, South Africa. Collins Chabane municipality covers an area of approximately 5003 km^2^ with an estimated population of 347,975. Research has shown that in the Soutpansberg region (which includes Vhembe district) factors such as weathering of silicates, ion exchange processes as well as dissolution of carbonates and halite minerals are hydro-geochemical processes which influence the quality of groundwater^7^. The main land use activities are residential settlements, agriculture and natural forests. Water supply from the municipality is inadequate in the study area as some of the households are not connected to the municipal line, thus, most of the residents rely on groundwater from boreholes. Furthermore, the study area has no established infrastructure for sewage systems and residents rely on pit latrines for sanitation.

## Material and methods

### Water sampling

**Figure 1:**
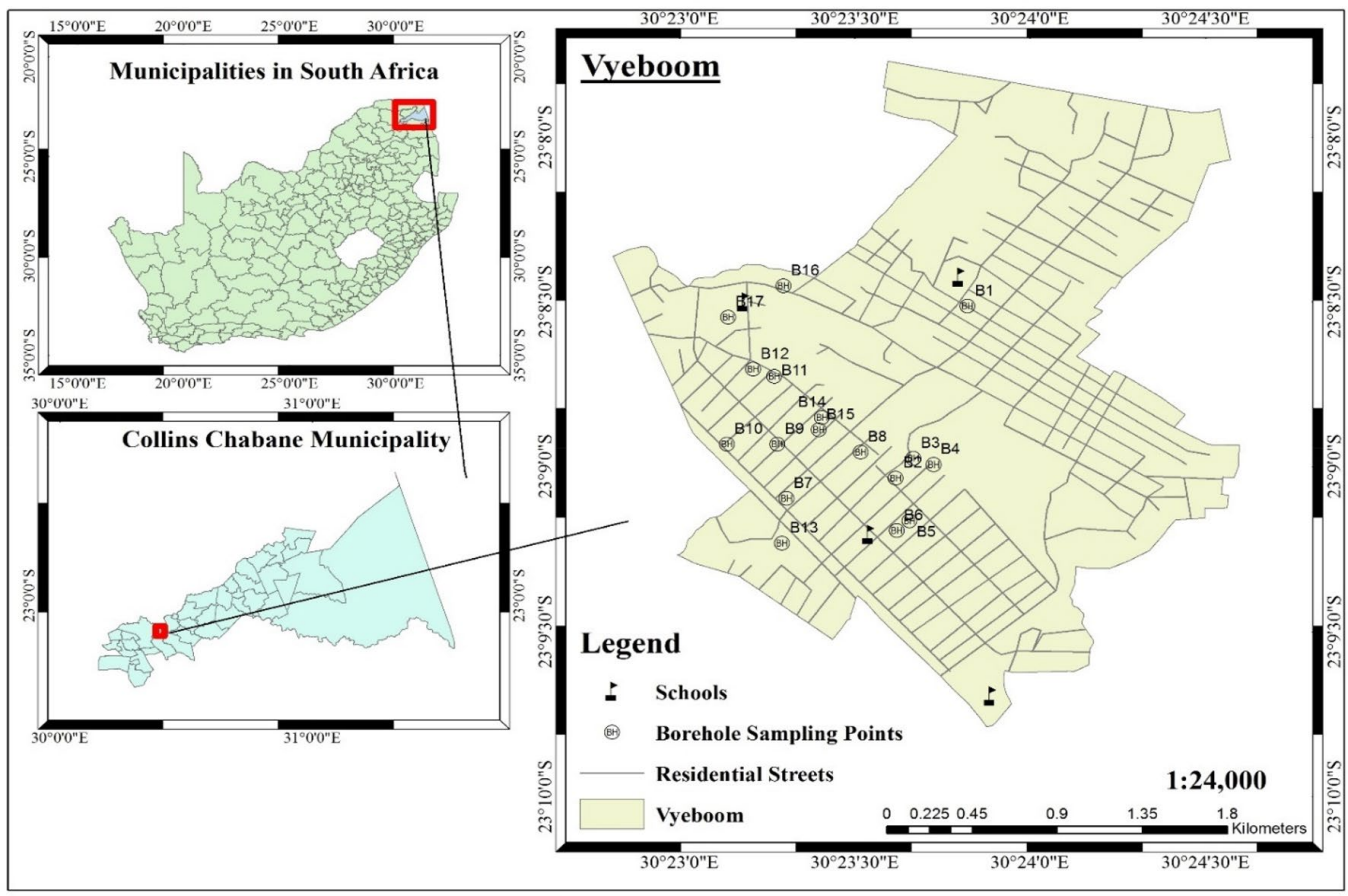
Map of the study area and the sampling points.

Water samples were collected from 17 randomly selected boreholes (14 private and 3 communal) at Vyeboom. The sampling point are represented in Fig. 1B1–B17 which shows the sampling points as well as the coordinates of the study area (230 9′ 00″ South and 300 23′ 00″ East).

Samples were collected in triplicates for physicochemical and microbiological parameters using sterile containers. Each sample was labeled according to the sampling point code (B1–B17). An Extech multimeter were used onsite to measure the electrical conductivity, salinity, pH and temperature of the samples. A turbidity meter was also used to measure turbidity of the water samples. Prior to measurements, the multimeters were calibrated in adherence to the manufacturers guidelines. After on-site sampling, samples were transported to the laboratory of the University of Venda for further analysis in ice-cold condition using a cooler box. Microbial parameters were analysed within 6 h of sample collection.

### Anions and trace metals analysis

A 45 ml volume of each water sample was transferred to a CEM MARS vessel followed by the addition of 5 ml nitric acid. The solution was mixed by swirling the vessel after which it was placed on the microwave digester which was set to operate at intervals of 10 min. After digestion was complete, the samples were allowed to cool for at least 15 min and then preserved at 4 °C. The digested samples were analysed using Inductively Coupled Plasma Optical Emission Spectrometer (ICP-OES). For anion analysis, the samples were filtered using a syringe filter and major anions such as chloride, fluoride, nitrate, and sulphate, were analysed using the Ion Chromatography method (IC).

### Microbiological analysis

The media used were m-Tech and m-Endo agars. For the preparation of m-Tech agar, 45.6 g of the agar was suspended in 1 L of distilled water and placed on a hot plate. The mixture was heated with a continuous stirring using a stir bar until the agar was completely dissolved^11^, after which it was sterilized by autoclaving at 121 °C for 15 min. Similarly, 51 g of m-Endo agar was added to 1 L distilled water and placed on the hot plate with continuous stirring until the agar is completely dissolved.

The membrane filtration method was used for the analysis of *E. coli* and total coliform. 100 ml of water sample was filtered through a sterile filter funnel containing a sterile 0.45 µm cellulose filter paper of 47 mm diameter. Membrane filters were then removed from the funnel using clamped forceps and transferred into a prepared petri-dish containing the prepared agars. The petri dishes were subsequently incubated at 37 °C for about 24 h. Afterwhich the number of bacterial colony forming units per 100 ml was counted and recorded^11^.

### Health risk assessment

Risk assessments have been estimated for ingestion and dermal pathways. Exposure pathways to water for ingestion and dermal routes are calculated using Eqs. 1 and 2^12,13^.

1 $${\mathrm{Exp}}_{\mathrm{ing}} =\frac{IR \times {C}_{water}\times EF \times ED}{AT \times BW}$$

2 $${\mathrm{Exp}}_{\mathrm{derm}}=\frac{{C}_{water} \times SA \times ET \times EF \times ED \times CF \times {K}_{p}}{AT \times BW}$$

where, Exp_ing_: exposure dose through ingestion of water (mg/kg/day); IR: ingestion rate in this study (2.2 L/day for adults; 1.8 L/day for children); C_water_: average concentration of the estimated metals in water (μg/L); EF: exposure frequency (365 days/year); ED: exposure duration (70 years for adults; and 6 years for children); AT: averaging time (365 days/year × 70 years for an adult; 365 days/year × 6 years for a child); BW: average body weight (70 kg for adults; 15 kg for children); Exp_derm_: exposure dose through dermal absorption (mg/kg/day); SA: exposed skin area (18,000 cm^2^ for adults; 6600 cm^2^ for children); ET: exposure time (0.58 h/ day for adults; 1 h/day for children); CF: unit conversion factor (0.001 L/cm^3^) and K_p_: dermal permeability coefficient in water, (cm/h), 0.001 for Cu, Mn, Fe and Cd, while 0.0006 for Zn; 0.002 for Cr and 0.004 for Pb^12,13^.

The hazard quotient (HQ) of non-carcinogenic risk by ingestion pathway can be determined by Eq. 3^14^. Where, RfD_ing_ is ingestion toxicity reference dose (mg/kg/day). A HQ below 1 is assumed to be safe and taken as significantly non-carcinogenic, but an HQ value above 1 may be a major potential health concern in association with over-exposure of humans to the contaminants.

3 $${\mathrm{HQ}}_{\mathrm{ing}/\mathrm{derm}} =\frac{{EXP}_{ing/derm}}{{RfD}_{ing/derm}}$$

The total non-carcinogenic risk is represented by the hazard index (HI). HI < 1 means the non-carcinogenic risk is acceptable, while HI > 1 indicates the risk is beyond the acceptable level^14^. The HI of a given pollutant through multiple pathways can be calculated by the summation of the hazard quotients based on Eq. 4^15^.

4 $$\mathrm{HI }=\sum_{i=1}^{n}{HQ}_{ing/derm}$$

Carcinogenic risks for the ingestion pathway are calculated by Eq. 5^16^. For the selected metals in the study, carcinogenic risk (CR_ing_) can be defined as the incremental probability that an individual will develop cancer during his lifetime due to exposure under specific scenarios^16^.

5 $${\mathrm{CR}}_{\mathrm{ing}} =\frac{{D}_{ing}}{{SF}_{ing}}$$

## Results and discussion

### Microbial contamination of water sources

**Figure 2:**
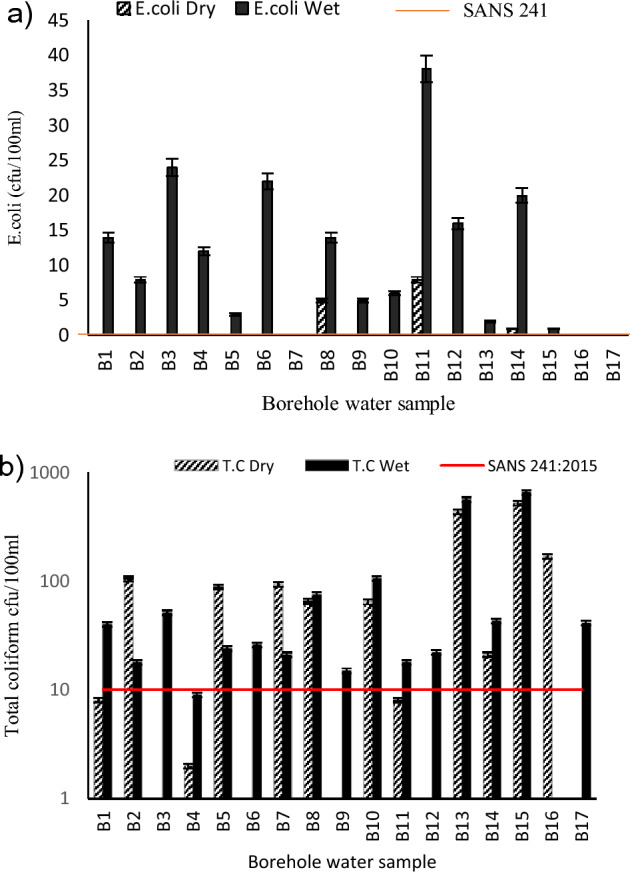
(**a**) *E.coli* counts of the water samples. (**b**)Total Coliform counts of the water samples.

*E. coli* is considered the most suitable indicator of feacal contamination^17^. Usually it occurs in high numbers in human and animal wastes, as well as in sewage^17^. Its presence in water sample indicates the presence of feacal material and the likelihood of harmful disease-causing pathogens. *E. coli* levels ranged from 0 to 8 cfu/100 mL in the dry season to 0–38 cfu/100 mL in the wet season (Fig. 2a). Higher levels were recorded in the wet season (87.5%) in boreholes B1, B2, B3, B4, B5, B6, B8, B9, B10, B11, B12, B13 and B14 than in the dry season (18.75%) in boreholes B8 and B11 and this could be due to the infiltration of contaminated water into the aquifer. Similarly, higher levels of total coliform (Fig. 2b) were also recorded in all boreholes(9–653 cfu/100 mL) except B4 and B 16 in the wet season, and in all boreholes except B1, B3, B4, B6, B9, B11, B12 and B17 in the dry season . Water borne diseases have been linked to the consumption of groundwater contaminated with faecal matter in previous studies conducted in similar setting globally^18^. This therefore insinuates that residents who consume water from the boreholes are at risk of infection. The World Health Organisation recommended 0 cfu/100 mL of *E. coli* for drinking water^17^ SANS recommends 0 cfu/100 ml for *E. coli* and 10 cfu/100 ml for total coliform^19^.

### Anions levels of groundwater samples from the study area

**Figure 3:**
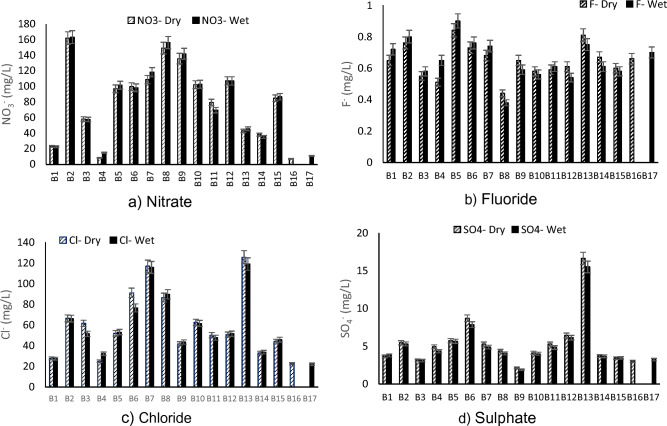
Anions concentrations for sampled boreholes in dry and wet season.

Nitrate is the final product of nitrogenous organic matter and a high concentration in water is an indication of water pollution. Groundwater quality associated with nitrate contamination has been widely reported^6^. The nitrate sources in groundwater are organic nitrogen and fertilizers used in agriculture^20^. The guideline value for nitrate is 50 mg/L (or 11 mg/L if reported as nitrate-nitrogen) according to the WHO^17^ and SANS^19^. High levels of nitrate has been linked with methaemoglobinaemia in infants, which is a result of short-term exposure^17^. The values of nitrate concentrations ranged from 7.29 to 163.14 mg/L during the study period (Fig. 3a). The highest values were obtained in B2, B8, and B9 with the values of 163.14 mg/L, 156.15 mg/L and 141.71 mg/L, respectively. Other sampled boreholes exceeded the recommended WHO standard of 50 mg/L except for B1, B4, B13, B14, B16 and B17. High concentrations of nitrate may be due to agricultural practices and the location of pit latrines which are located near the boreholes^21^. The levels of nitrate recorded in some of the boreholes is of public health concern as it may negatively impact the consumers.

Traces of fluorides (F^-^) are present in many waters with higher concentrations often associated with groundwater. F^−^ is the most electronegative and reactive element on earth and is beneficial to human health in trace amounts (0.5– < 1.5 mg/L)^22,23^. The obtained values of Fluoride ranged from (0.44–0.9 mg/L) during the sampling period (Fig. 3b). High levels of fluoride can pose a human health problem by causing skeletal fluorosis^24^. The study revealed that all boreholes complied with the recommended standards of < 1.5 mg/L set by SANS^19^. Higher levels of fluoride has been reported from groundwater samples from Siloam village, Makhado local municipality in the same province of South Africa^25^. Similarly the East African Rift system are known for high levels of fluoride in groundwater^3^.

Chloride concentrations of over 250 mg/L can impart taste to groundwater^17^. Higher levels of chloride have been reported in other parts of the country and the world^3^. The samples in this study recorded low to medium levels of chloride 22.16–125.45 mg/L (Fig. 3c) which complied to the nations regulatory guideline value of < 300 mg/L^19^.

Sulphate is another component that exists in drinking water naturally. Health concerns such as diarrhoea have been linked to consumption of water with high levels of sulphate. The presence of sulphates in water can be characterised with a bitter taste especially at concentration > 250 mg/L^17^, thus making it unpleasant for drinking purposes. High sulphate concentration may cause respiratory problems in humans^26^. The amount of sulphate recorded in this study ranged from 1.92–16.62 mg/L (Fig. 3d) and it complied with the recommended standard (200 mg/L) for domestic use set by SANS^19^.

### Cations levels of groundwater in the study area

The taste threshold concentration of sodium in water depends on the associated anion and the temperature of the solution. At room temperature, the average taste threshold for sodium is about 200 mg/L^17^ while the level of sodium recorded in the boreholes ranged between 28.1 and 52.5 mg/L (Table 1) which was within the recommended SANS standard of < 200 mg/L^19^. Potassium is also an essential element in humans and is seldom found in drinking water at levels that could be a concern to health^17^. The mean concentration of potassium in the water samples ranged from 0.88 to 2.94 mg/L (Table 3) which was within WHO^17^ and SANS^19^ acceptable limits.

**Table 1:** The concentrations of major cations during in dry and wet period.

Sample ID	Sodium (mg/L)		Potassium (mg/L)		Magnesium (mg/L)		Calcium (mg/L)	
	Dry	Wet	Dry	Wet	Dry	Wet	Dry	Wet
B1	28.10	38.20	2.56	2.49	18.40	18.40	63.40	75.30
B2	52.50	51.20	1.00	0.94	24.30	24.60	60.80	61.10
B3	32.60	32.80	1.14	1.16	19.60	19.80	57.00	57.90
B4	31.50	31.50	1.17	1.12	16.80	17.10	57.50	58.50
B5	40.50	39.70	0.94	0.88	21.00	21.20	76.50	76.90
B6	43.30	41.20	1.00	0.98	28.00	26.00	99.70	93.70
B7	51.30	50.20	1.23	1.19	31.90	30.60	81.30	78.50
B8	50.90	49.00	1.53	1.46	32.10	30.80	78.80	78.30
B9	44.10	42.10	1.90	1.88	17.50	17.10	58.00	57.30
B10	41.40	39.50	1.91	1.85	22.40	21.80	67.70	66.80
B11	41.90	40.10	2.94	2.74	22.40	21.10	75.40	72.20
B12	46.10	44.80	2.82	2.73	20.90	20.30	68.80	67.90
B13	43.30	41.40	1.10	1.14	32.30	30.10	67.10	64.20
B14	38.20	37.00	2.18	2.20	19.80	19.50	49.90	58.20
B15	37.80	40.40	1.67	1.74	21.10	20.60	60.40	61.10
B16	47.50	Na	2.12	Na	17.10	Na	45.70	Na
B17	Na	40.30	Na	2.37	Na	18.10	Na	65.50
Min	28.10	31.50	0.94	0.88	16.80	17.10	45.70	57.30
Max	52.50	51.20	2.94	2.74	32.30	30.80	99.70	93.70
Ave	41.94	41.21	1.70	1.68	22.85	22.32	66.75	68.34
St.Dev	6.88	5.33	0.65	0.64	5.21	4.55	12.96	9.83

The concentrations of Mg ranged between 16.8 to 32.3 mg/L which is in compliance with the WHO recommendation of 50 mg/L for domestic use^27^. Ordinarily, high magnesium concentration is associated with run-off effluents and leaching from agricultural activities^28^. According to WHO^17^, Ca is vital for biochemical interactions in all living organisms. The obtained values of Ca ranged between 45.7 and 99.7 mg/L and complied to regulatory guideline (Table 1).

### Groundwater hydrochemistry

**Figure 4:**
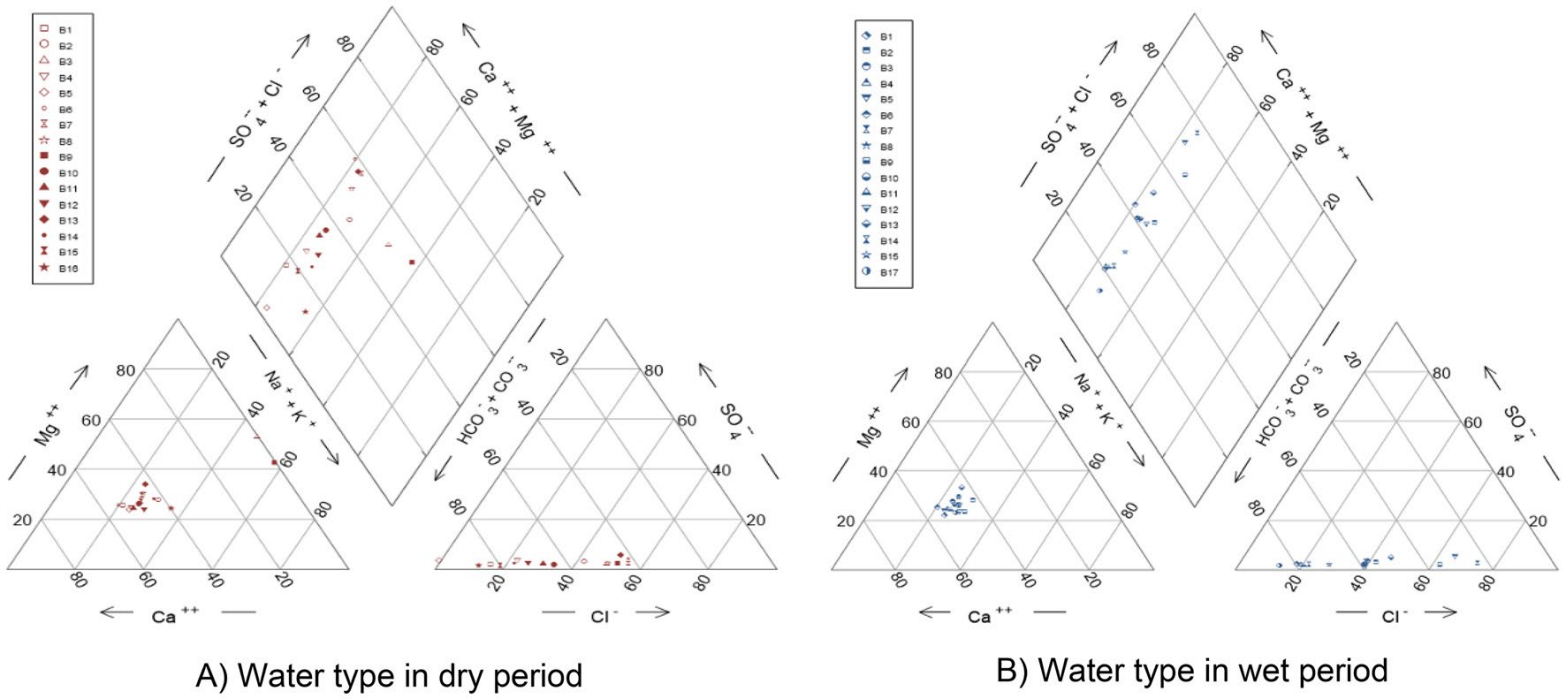
Piper diagram showing the water type of boreholes for both dry and wet periods.

The major ions in groundwater within the study area was plotted on the Piper trilinear plot. The plot consist of a triangle plots for cations and anions respectively. The diamond plot was for the combined indication of the water type^29^. From the Piper plot in both the wet and the dry season, no dominant type cation was predominant in both seasons except a few samples having Ca^2+^ as the dominant cation, whereas bicarbonate HCO_3_^−^ was the major anion recorded for both season (Fig. 4). From the plot it can be concluded that the Mg(HCO_3_)_2_ water type was predominant in the study area and this implies that alkaline earth metals (Ca + Mg) exceeds alkali metals (Na + K) in the aquifers of most of the boreholes in the study area. Also the weak acid (HCO_3_) dominates the strong acids (SO_4_ and Cl). Few of the borehole samples recorded calcium chloride water type mostly in the wet season.

### Physicochemical parameters

Electrical conductivity (EC) is a measurement of the ease with which water conducts electricity and can be used as a proxy for salinity and TDS determination in water^24^. The average values of EC in the samples ranged from 47.2 to 85 mS/m (Table 2). EC values were greater in the wet season compared to the dry in all the sampling points which could be due to increased infiltration of water and ionic materials during the wet season. Furthermore, there could be increased leaching of ions from host rocks due to their interaction with more water in the wet season^30^. According to the standard set by SANS (< 170 mS/m), EC in all the boreholes complied with the recommended standard^19^. Average values of salinity ranged from 250 to 446 mg/L (Table 2) and higher levels were recorded in the wet season.

**Table 2:** Physicochemical variation in the dry and wet season.

Sample ID	EC (mS/m)		Temp (°C)		pH		Salinity (mg/L)	
	Dry	Wet	Dry	Wet	Dry	Wet	Dry	Wet
B1	58.60	58.60	24.30	27.70	7.58	7.58	305.50	313.00
B2	69.10	70.00	25.05	27.80	7.57	7.40	358.00	369.50
B3	53.30	53.60	25.50	27.65	7.01	7.24	277.50	284.00
B4	47.20	49.50	24.25	27.45	7.23	7.38	250.00	263.00
B5	65.80	66.00	23.95	27.50	7.59	7.81	345.00	347.50
B6	83.80	78.20	24.20	27.45	7.03	7.34	446.00	416.50
B7	85.00	83.10	24.60	27.40	7.21	7.12	441.50	438.00
B8	82.20	80.00	24.75	27.65	7.18	7.33	427.50	440.50
B9	58.80	57.00	24.35	27.20	7.31	7.29	303.00	378.00
B10	64.30	63.10	24.80	27.15	7.68	7.34	336.50	343.00
B11	65.20	61.70	25.10	27.80	7.34	7.45	339.00	333.50
B12	66.10	63.70	24.15	27.85	7.32	7.14	350.50	347.00
B13	72.90	69.00	24.25	27.45	7.80	8.05	390.50	377.50
B14	53.80	55.00	24.50	27.70	7.35	7.95	300.50	299.50
B15	58.10	56.80	25.00	27.70	7.19	7.29	348.50	309.50
B16	52.50	Na	25.25	Na	7.57	Na	279.50	Na
B17	Na	51.60	Na	27.70	Na	7.76	Na	283.00
Min	47.20	49.50	23.95	27.15	7.01	7.12	250.00	263.00
Max	85.00	83.10	25.50	27.85	7.80	8.05	446.00	440.50
Average	64.79	63.56	24.63	27.57	7.37	7.47	343.69	346.44
St.dev	11.16	9.92	0.44	0.20	0.23	0.27	56.93	52.67

Na represent no access.

Temperature influences the state of different inorganic components and chemical pollutants that may impair taste^31,32^. High water temperature also enhances micro-organism growth and can increase taste, odor, color and corrosion-related problems^17^. The average temperatures in all selected sampling points ranged between 23.95 and 27.85 °C (Table 2) which fell within the recommended guideline.

The pH of groundwater in the study area also ranged from 7.01 to 8.05 (Table 2) throughout the sampling period. These values were within the SANS recommendation of 6–9 for human consumption^19^. Turbidity is caused by clay, silt, fine organic and inorganic matter as well as plankton and micro-organisms suspended in water. Most of the samples had very low turbidity values which shows good asthethic property of the groundwater in compliance with SANS guidleine.

### Trace metals concentrations in groundwater

**Figure 5:**
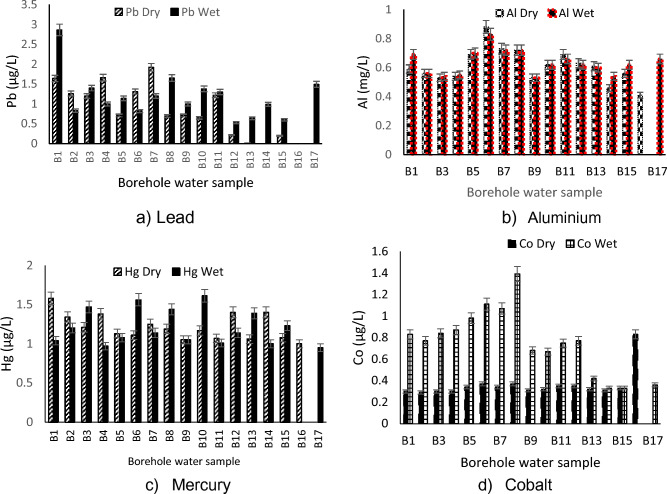
Levels of Pb, Hg, Al and Co in the dry and wet season of the study area.

The mean concentrations of Lead (Pb) obtained ranged from < 0.05 to 2.86 µg/L (Fig. 5a) during the dry and wet periods which was below the SANS regulatory standards of < 10 µg/L. Pb is highly toxic and can lead to health problems such as feotal tissue damage, fever, anxiety, abdominal pain, nerve damage, kidney, brain and liver damage, blood pressure, lung and stomach cancer, hereditary behavioural disorders, reproductive impairment and anaemia^33^. According to WHO^17^ Pb is used principally in the production of Lead-acid batteries, solder and alloys. Exposure to high concentration could also cause kidney and brain damage in males^34^. Water with less than 5 µg/L concentration of Pb could have the possibility of neurological impairment in foetuses as well as brain impairment in young children during developing stages^28^.

The average levels of Aluminium (Al) ranged between 0.41 and 0.88 mg/L (Fig. 5b). All boreholes did not comply with the operational standard limit set by SANS^19^ of 0.3 mg/L. At levels exceeding the threshold limit, Al can impact unpleasant tast to water and affect its asthetic property. The levels of Al recorded may be from the geogenic material in the aquifer where groundwater accumulates. Alzheimer’s disease and renal failure have been linked with the consumption of Al groundwater from boreholes^35^.

Mercury is present in inorganic form in surface water and groundwater at concentrations usually below < 0.5 μg/l, although local mineral deposits may produce higher levels in groundwater^17^. Concentrations of mercury ranged between 0.95 and 1.61 µg/L during the sampling period (Fig. 5c). All boreholes were within the recommended standard set by SANS^19^ and WHO^17^ of < 6 µg/L. Throughout all the sampling points, low levels of Co was also recorded during the study period which complied to the SANS regulatory standard (Fig. 5d).

**Figure 6:**
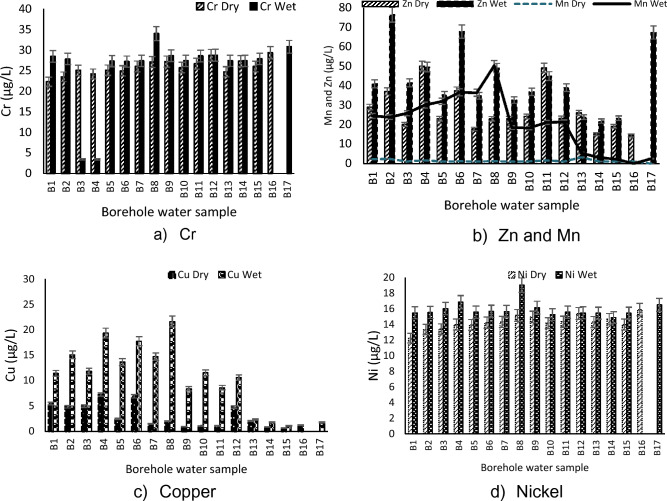
Level of heavy metals concentration throughout the study period.

Consumption of water with Cr concentration greater than 0.05 mg/L (50 µg/L) has a possible risk of causing gastrointestinal cancer with long-term exposure, undesirable taste and slight nausea in humans^28^. Chromium concentrations recorded in this study were in the range of 3.42–34.01 µg/L which is acceptable for domestic use (Fig. 6a).

Zinc is another essential trace element found in virtually all food and potable water in the form of salts or organic complexes. Zinc imparts an undesirable taste to water at a threshold concentration of about 4 mg/L (as Zinc Sulphate). Water containing Zn at concentrations in excess of 3–5 mg/L may appear opalescent and develop a greasy film on boiling ^17^. According to Asare-Donkor et al*.*^36^, Zn is known to have antioxidant properties that protect humans against accelerated aging of muscles and skin. It also helps in the healing process after an injury if a moderate and recommended dosage is ingested. The average values of Zn recorded ranged between 14.43 and 76.40 µg/L (Fig. 6b) and all the boreholes complied with the SANS standard of < 5000 µg/L.

At levels exceeding 0.1 mg/L, Mn causes undesirable taste in beverages. Results obtained (Fig. 6b) showed a concentration range of 0.97–50.33 µg/L as well as a sudden increase in concentration during the wet season which may be caused by rainwater. The results also showed that all boreholes complied with the recommendation (SANS)^19^ of < 400 µg/L for health effects and < 100 µg/L for aesthetic effects.

Copper is both an essential nutrient and a drinking-water contaminant. Staining of laundry and sanitary ware occurs at concentrations above 1000 µg/L. At levels above 2.5 mg/L, Copper imparts an undesirable bitter taste to water, at higher levels, the colour of the water is also altered^17^ and is associated with cardiovascular complications^28^. The recorded values of Cu ranged between 0.68 and 21.31 µg/L (Fig. 6c). This study revealed that the concentration of copper drastically increased in the wet season. All boreholes complied with the recommended standard of < 2000 µg/L set by SANS^19^.

According to WHO^17^, Nickel released from taps and fittings may contribute up to 1 mg/L in special cases of release from natural or industrial Nickel deposits. The average concentrations of Nickel detected in this study ranged between 12.24 and 19.01 µg/L (Fig. 6d) during the sampling duration and all the boreholes (concentrations) fell within the standard set by SAN^19^.

### Human health risk assessment

The potential human health risk associated with the consumption of water from the study area were computed using the parameters in Eqs. 1–5 and based on the data presented in Tables 3, 4 and 5 below. The estimated non carcinogenic risk were < 1 for the dermal route using the minimum, average and maximum levels of trace metals in the borehole of the study area. This implies that the use of the groundwater for bathing purposes shows no lifetime risk to the users. Similar findings have been reported both within and outside of South Africa^37^. Exposure due to the ingestion also showed no non carcinogenic risk associated with the consumption of the water resource by adults. This was confirmed with the computation of the hazard quotient (HQ) and the hazard index (HI) which is a sum of both exposure routes. However, some of the values were close to 1 indicating a possibility of the non-carcinogenic risk of the water changing over time due to either geonic or anthropogenic sources of pollution. Furthermore, the exposure risk for the ingestion pathway for children revealed the possibility of having non-carcinogenic health risk. Though there is no risk associated with exposure to the minimum levels of the metals, the mean and the maximum levels showed the possibility as the summation of the risk from both the dermal and the ingestion pathways was greater than 1. It is noteworthy to state that although individual metal may not pose risk due to the levels recorded in the study area, the synergistic effects of the metals in the water resources do pose a risk.

**Table 3:** Dermal exposure of (a) adults and (b) children to trace metals in groundwater from the study area in the dry and wet season.

Metals	Metal conc (µg/L)			EXP_derm_			RF_derm_	HQ_derm_		
	Min	Avg	max	Min	Avg	Max		Min	Avg	Max
(a)										
Dry season										
Al	10.00	791.00	1660.00	1.49E-03	1.18E-01	2.48E-01	140.00	1.07E-05	8.43E-04	1.77E-03
Pb	0.01	0.84	1.64	5.97E-06	5.01E-04	9.78E-04	0.42	1.42E-05	1.19E-03	2.33E-03
Hg	1.00	1.21	1.58	5.97E-04	7.24E-04	9.43E-04	0.40	1.49E-03	1.81E-03	2.36E-03
Cr	22.29	25.86	29.35	6.65E-03	7.71E-03	8.75E-03	0.08	8.87E-02	1.03E-01	1.17E-01
Zn	14.43	26.94	50.08	1.29E-02	2.41E-02	4.48E-02	60	2.15E-04	4.02E-04	7.47E-04
Mn	0.97	1.46	3.25	1.45E-04	2.18E-04	4.85E-04	0.96	1.51E-04	2.27E-04	5.05E-04
Cu	0.68	3.02	7.28	1.01E-04	4.51E-04	1.09E-03	12.00	8.45E-06	3.76E-05	9.05E-05
Co	0.29	0.36	0.83	4.33E-05	5.34E-05	1.24E-04	5.40	8.01E-06	9.89E-06	2.29E-05
Ni	12.24	14.26	15.88	7.30E-03	4.25E-03	4.74E-03	5.40	1.35E-03	7.87E-04	8.77E-04
Wet season										
Al	600.00	1111.00	2860.00	8.95E-02	1.66E-01	4.27E-01	140.00	6.39E-04	1.18E-03	3.05E-03
Pb	0.60	1.18	2.86	3.58E-04	7.02E-04	1.71E-03	0.42	8.52E-04	1.67E-03	4.06E-03
Hg	0.95	1.21	1.61	5.67E-04	7.19E-04	9.60E-04	0.40	1.42E-03	1.80E-03	2.40E-03
Cr	3.42	25.35	34.01	1.02E-03	7.56E-03	1.01E-02	0.08	1.36E-02	1.01E-01	1.35E-01
Zn	21.83	42.79	67.27	1.95E-02	3.83E-02	6.02E-02	60.00	3.26E-04	6.38E-04	1.00E-03
Mn	2.25	21.99	50.33	3.36E-04	3.28E-03	7.51E-03	0.96	3.50E-04	3.42E-03	7.82E-03
Cu	1.81	10.73	21.61	2.70E-04	1.60E-03	3.22E-03	12.00	2.25E-05	1.33E-04	2.69E-04
Co	0.33	0.76	1.39	4.92E-05	1.13E-04	2.07E-04	5.40	9.11E-06	2.10E-05	3.84E-05
Ni	14.87	15.89	19.01	8.87E-03	9.48E-03	1.13E-02	5.40	1.64E-03	1.76E-03	2.10E-03
(b)										
Dry season										
Al	10.00	791.00	1660.00	3.77E-04	3.48E-01	7.30E-01	140.00	2.69E-06	2.49E-03	5.22E-03
Pb	0.01	0.84	1.64	1.51E-06	3.70E-04	7.22E-04	0.42	3.59E-06	8.80E-04	1.72E-03
Hg	1.00	1.21	1.58	1.51E-04	5.34E-04	6.95E-04	0.40	3.77E-04	1.33E-03	1.74E-03
Cr	22.29	25.86	29.35	1.68E-03	1.14E-02	1.29E-02	0.08	2.24E-02	1.52E-01	1.72E-01
Zn	14.43	26.94	50.08	3.27E-03	1.19E-02	2.20E-02	60.00	5.44E-05	1.98E-04	3.67E-04
Mn	0.97	1.46	3.25	3.66E-05	6.42E-04	1.43E-03	0.96	3.81E-05	6.69E-04	1.49E-03
Cu	0.68	3.02	7.28	2.56E-05	1.33E-03	3.20E-03	12.00	2.14E-06	1.11E-04	2.67E-04
Co	0.29	0.36	0.83	1.09E-05	1.58E-04	3.65E-04	5.40	2.03E-06	2.92E-05	6.76E-05
Ni	12.24	14.26	15.88	1.85E-03	6.27E-03	6.99E-03	5.40	3.42E-04	1.16E-03	1.29E-03
Wet season										
Al	600.00	1111.00	2860.00	2.26E-02	4.89E-01	1.26E + 00	140.00	1.62E-04	3.49E-03	8.99E-03
Pb	0.60	1.18	2.86	4.53E-05	2.07E-03	5.03E-03	0.42	1.08E-04	4.93E-03	1.20E-02
Hg	0.95	1.21	1.61	7.17E-05	2.12E-03	2.83E-03	0.40	1.79E-04	5.30E-03	7.08E-03
Cr	3.42	25.35	34.01	2.58E-04	2.23E-02	2.99E-02	0.08	3.44E-03	2.97E-01	3.99E-01
Zn	21.83	42.79	67.27	4.94E-03	1.13E-01	1.78E-01	60.00	8.23E-05	1.88E-03	2.96E-03
Mn	2.25	21.99	50.33	8.49E-05	9.68E-03	2.21E-02	0.96	8.84E-05	1.01E-02	2.31E-02
Cu	1.81	10.73	21.61	6.83E-05	4.72E-03	9.51E-03	12.00	5.69E-06	3.93E-04	7.92E-04
Co	0.33	0.76	1.39	1.24E-05	3.35E-04	6.12E-04	5.40	2.30E-06	6.20E-05	1.13E-04
Ni	14.87	15.89	19.01	2.24E-03	2.80E-02	3.35E-02	5.40	4.15E-04	5.18E-03	6.20E-03

**Table 4:** Ingestion exposure of (a) adults and (b) children to trace metals in groundwater from the study area in the dry and wet season.

Metals	Metal conc (µg/L)			EXP_ing_			RF_ing_	HQ_ing_		
	Min	Avg	max	min	avg	max		min	avg	Max
(a)										
Dry season										
Al	10.00	791.00	1660.00	3.24E-01	2.26E + 01	4.74E + 01	700.00	4.62E-04	3.23E-02	6.78E-02
Pb	0.01	0.84	1.64	3.24E-04	2.40E-02	4.69E-02	1.40	2.31E-04	1.71E-02	3.35E-02
Hg	1.00	1.21	1.58	3.24E-02	3.47E-02	4.51E-02	0.30	1.08E-01	1.16E-01	1.50E-01
Cr	22.29	25.86	29.35	7.22E-01	7.39E-01	8.39E-01	3.00	2.41E-01	2.46E-01	2.80E-01
Zn	14.43	26.94	50.08	4.67E-01	7.70E-01	1.43E + 00	300.00	1.56E-03	2.57E-03	4.77E-03
Mn	0.97	1.46	3.25	3.14E-02	4.17E-02	9.29E-02	140.00	2.24E-04	2.98E-04	6.63E-04
Cu	0.68	3.02	7.28	2.20E-02	8.64E-02	2.08E-01	40.00	5.50E-04	2.16E-03	5.20E-03
Co	0.29	0.36	0.83	9.39E-03	1.02E-02	2.37E-02	20.00	4.69E-04	5.11E-04	1.19E-03
Ni	12.24	14.26	15.88	3.96E-01	4.07E-01	4.54E-01	20.00	1.98E-02	2.04E-02	2.27E-02
Wet season										
Al	600.00	1111.00	2860.00	1.71E + 01	3.17E + 01	8.17E + 01	700.00	2.45E-02	4.53E-02	1.17E-01
Pb	0.60	1.18	2.86	1.71E-02	3.36E-02	8.17E-02	3.50	4.90E-03	9.61E-03	2.33E-02
Hg	0.95	1.21	1.61	2.71E-02	3.44E-02	4.60E-02	0.30	9.05E-02	1.15E-01	1.53E-01
Cr	3.42	25.35	34.01	9.77E-02	7.24E-01	9.72E-01	3.00	3.26E-02	2.41E-01	3.24E-01
Zn	21.83	42.79	67.27	6.24E-01	1.22E + 00	1.92E + 00	300.00	2.08E-03	4.08E-03	6.41E-03
Mn	2.25	21.99	50.33	6.43E-02	6.28E-01	1.44E + 00	140.00	4.59E-04	4.49E-03	1.03E-02
Cu	1.81	10.73	21.61	5.17E-02	3.07E-01	6.17E-01	40.00	1.29E-03	7.66E-03	1.54E-02
Co	0.33	0.76	1.39	9.43E-03	2.17E-02	3.97E-02	20.00	4.71E-04	1.09E-03	1.99E-03
Ni	14.87	15.89	19.01	4.25E-01	4.54E-01	5.43E-01	20.00	2.12E-02	2.27E-02	2.72E-02
(b)										
Dry season										
Al	10.00	791.00	1660.00	6.67E-01	5.27E + 01	1.11E + 02	700.00	9.52E-04	7.53E-02	1.58E-01
Pb	0.01	0.84	1.64	6.67E-04	5.60E-02	1.09E-01	3.50	1.90E-04	1.60E-02	3.12E-02
Hg	1.00	1.21	1.58	6.67E-02	8.09E-02	1.05E-01	0.30	2.22E-01	2.70E-01	3.51E-01
Cr	22.29	25.86	29.35	1.49E + 00	1.72E + 00	1.96E + 00	3.00	4.95E-01	5.75E-01	6.52E-01
Zn	14.43	26.94	50.08	9.62E-01	1.80E + 00	3.34E + 00	300.00	3.21E-03	5.99E-03	1.11E-02
Mn	0.97	1.46	3.25	6.47E-02	9.73E-02	2.17E-01	140.00	4.62E-04	6.95E-04	1.55E-03
Cu	0.68	3.02	7.28	4.53E-02	2.02E-01	4.85E-01	40.00	1.13E-03	5.04E-03	1.21E-02
Co	0.29	0.36	0.83	1.93E-02	2.39E-02	5.53E-02	20.00	9.67E-04	1.19E-03	2.77E-03
Ni	12.24	14.26	15.88	8.16E-01	9.50E-01	1.06E + 00	20.00	4.08E-02	4.75E-02	5.29E-02
Wet season										
Al	600.00	1111.00	2860.00	4.00E + 01	7.41E + 01	1.91E + 02	700.00	5.71E-02	1.06E-01	2.72E-01
Pb	0.60	1.18	2.86	4.00E-02	7.85E-02	1.91E-01	3.50	1.14E-02	2.24E-02	5.45E-02
Hg	0.95	1.21	1.61	6.33E-02	8.03E-02	1.07E-01	0.30	2.11E-01	2.68E-01	3.58E-01
Cr	3.42	25.35	34.01	2.28E-01	1.69E + 00	2.27E + 00	3.00	7.60E-02	5.63E-01	7.56E-01
Zn	21.83	42.79	67.27	1.46E + 00	2.85E + 00	4.48E + 00	300.00	4.85E-03	9.51E-03	1.49E-02
Mn	2.25	21.99	50.33	1.50E-01	1.47E + 00	3.36E + 00	140.00	1.07E-03	1.05E-02	2.40E-02
Cu	1.81	10.73	21.61	1.21E-01	7.15E-01	1.44E + 00	40.00	3.02E-03	1.79E-02	3.60E-02
Co	0.33	0.76	1.39	2.20E-02	5.07E-02	9.27E-02	20.00	1.10E-03	2.54E-03	4.63E-03
Ni	14.87	15.89	19.01	9.91E-01	1.06E + 00	1.27E + 00	20.00	4.96E-02	5.30E-02	6.34E-02

**Table 5:** The minimum, mean and maximum hazard quotients values for (a) children and (b) adult in the dry and wet seasons of the study area.

Metals	Minimum values			Average values			Maximum values		
	HQ_ing_	HQ_derm_	HI	HQ_ing_	HQ_derm_	HI	HQ_ing_	HQ_derm_	HI
(a)									
Dry season									
Al	9.52E-04	2.69E-06	9.55E-04	7.53E-02	2.49E-03	7.78E-02	1.58E-01	5.22E-03	1.63E-01
Pb	1.90E-04	3.59E-06	1.94E-04	1.60E-02	8.80E-04	1.69E-02	3.12E-02	1.72E-03	3.30E-02
Hg	2.22E-01	3.77E-04	2.23E-01	2.70E-01	1.33E-03	2.71E-01	3.51E-01	1.74E-03	3.53E-01
Cr	4.95E-01	2.24E-02	5.18E-01	5.75E-01	1.52E-01	7.26E-01	6.52E-01	1.72E-01	8.24E-01
Zn	3.21E-03	5.44E-05	3.26E-03	5.99E-03	1.98E-04	6.18E-03	1.11E-02	3.67E-04	1.15E-02
Mn	4.62E-04	3.81E-05	5.00E-04	6.95E-04	6.69E-04	1.36E-03	1.55E-03	1.49E-03	3.04E-03
Cu	1.13E-03	2.14E-06	1.14E-03	5.04E-03	1.11E-04	5.15E-03	1.21E-02	2.67E-04	1.24E-02
Co	9.67E-04	2.03E-06	9.69E-04	1.19E-03	2.92E-05	1.22E-03	2.77E-03	6.76E-05	2.83E-03
Ni	4.08E-02	3.42E-04	4.11E-02	4.75E-02	1.16E-03	4.87E-02	5.29E-02	1.29E-03	5.42E-02
Sum	7.65E-01	2.32E-02	7.89E-01	9.96E-01	1.59E-01	1.15E + 00	1.27E + 00	1.84E-01	1.46E + 00
Wet season									
Al	5.71E-02	1.62E-04	5.73E-02	1.06E-01	3.49E-03	1.09E-01	2.72E-01	8.99E-03	2.81E-01
Pb	1.14E-02	1.08E-04	1.15E-02	2.24E-02	4.93E-03	2.73E-02	5.45E-02	1.20E-02	6.65E-02
Hg	2.11E-01	1.79E-04	2.11E-01	2.68E-01	5.30E-03	2.73E-01	3.58E-01	7.08E-03	3.65E-01
Cr	7.60E-02	3.44E-03	7.94E-02	5.63E-01	2.97E-01	8.61E-01	6.39E-01	3.37E-01	9.76E-01
Zn	4.85E-03	8.23E-05	4.93E-03	9.51E-03	1.88E-03	1.14E-02	1.49E-02	2.96E-03	1.79E-02
Mn	1.07E-03	8.84E-05	1.16E-03	1.05E-02	1.01E-02	2.06E-02	2.40E-02	2.31E-02	4.70E-02
Cu	3.02E-03	5.69E-06	3.02E-03	1.79E-02	3.93E-04	1.83E-02	3.60E-02	7.92E-04	3.68E-02
Co	1.10E-03	2.30E-06	1.10E-03	2.54E-03	6.20E-05	2.60E-03	4.63E-03	1.13E-04	4.75E-03
Ni	4.96E-02	4.15E-04	5.00E-02	5.30E-02	5.18E-03	5.82E-02	6.34E-02	6.20E-03	6.96E-02
Sum	4.15E-01	4.48E-03	4.20E-01	1.05E + 00	3.29E-01	1.38E + 00	1.47E + 00	3.98E-01	1.86E + 00
(b)									
Dry season									
Al	4.62E-04	1.07E-05	4.73E-04	3.23E-02	8.43E-04	3.31E-02	6.78E-02	1.77E-03	6.95E-02
Pb	2.31E-04	1.42E-05	2.45E-04	1.71E-02	1.19E-03	1.83E-02	3.35E-02	2.33E-03	3.58E-02
Hg	1.08E-01	1.49E-03	1.09E-01	1.16E-01	1.81E-03	1.17E-01	1.50E-01	2.36E-03	1.53E-01
Cr	2.41E-01	8.87E-02	3.29E-01	2.46E-01	1.03E-01	3.49E-01	2.80E-01	1.17E-01	3.96E-01
Zn	1.56E-03	2.15E-04	1.77E-03	2.57E-03	4.02E-04	2.97E-03	4.77E-03	7.47E-04	5.52E-03
Mn	2.24E-04	1.51E-04	3.75E-04	2.98E-04	2.27E-04	5.25E-04	6.63E-04	5.05E-04	1.17E-03
Cu	5.50E-04	8.45E-06	5.59E-04	2.16E-03	3.76E-05	2.20E-03	5.20E-03	9.05E-05	5.29E-03
Co	4.69E-04	8.01E-06	4.77E-04	5.11E-04	9.89E-06	5.21E-04	1.19E-03	2.29E-05	1.21E-03
Ni	1.98E-02	1.35E-03	2.12E-02	2.04E-02	7.87E-04	2.12E-02	2.27E-02	8.77E-04	2.36E-02
Sum	3.72E-01	9.19E-02	4.64E-01	4.37E-01	1.08E-01	5.45E-01	5.66E-01	1.25E-01	6.91E-01
Wet season									
Al	2.45E-02	6.39E-04	2.51E-02	4.53E-02	1.18E-03	4.65E-02	1.17E-01	3.05E-03	1.20E-01
Pb	4.90E-03	8.52E-04	5.75E-03	9.61E-03	1.67E-03	1.13E-02	2.33E-02	4.06E-03	2.74E-02
Hg	9.05E-02	1.42E-03	9.19E-02	1.15E-01	1.80E-03	1.17E-01	1.53E-01	2.40E-03	1.56E-01
Cr	3.26E-02	1.36E-02	4.62E-02	2.41E-01	1.01E-01	3.42E-01	3.24E-01	1.35E-01	4.59E-01
Zn	2.08E-03	3.26E-04	2.40E-03	4.08E-03	6.38E-04	4.71E-03	6.41E-03	1.00E-03	7.41E-03
Mn	4.59E-04	3.50E-04	8.09E-04	4.49E-03	3.42E-03	7.90E-03	1.03E-02	7.82E-03	1.81E-02
Cu	1.29E-03	2.25E-05	1.32E-03	7.66E-03	1.33E-04	7.80E-03	1.54E-02	2.69E-04	1.57E-02
Co	4.71E-04	9.11E-06	4.81E-04	1.09E-03	2.10E-05	1.11E-03	1.99E-03	3.84E-05	2.02E-03
Ni	2.12E-02	1.64E-03	2.29E-02	2.27E-02	1.76E-03	2.45E-02	2.72E-02	2.10E-03	2.93E-02
Sum	1.78E-01	1.89E-02	1.97E-01	4.51E-01	1.11E-01	5.63E-01	6.79E-01	1.56E-01	8.35E-01

The findings from this study has revealed that some boreholes in the study area have high levels of nitrates, *E.coli*, total coliform and aluminium which have been discussed in Sects. “Microbial contamination of water sources”, “Anions levels of groundwater samples from the study area” and “Trace metals concentrations in groundwater”. Groundwater quality from these points are compromised and therefore possess health threats to residents mostly children.

The cancer risk was computed for two of the metals (Cr and Pb) which are believed to be carcinogens. Although mercury is a known carcinogen, the absence of a slope factor at the moment impeded the computation of its risk. However, the data derived from the other two carcinogens can be used as a proxy indicator for mercury. Subsequently, for both children and adults, the maximum values of Cr and Pb were greater than carcinogenic indices of 10^−4^ and 10^−6^ in both dry and wet seasons. Thus, the levels of Cr and Pb in the water showed the possibility of cancer risk with a lifetime consumption (Table 6). Although the possibility of drinking this water for a life time is slim, especially the younger population who will leave for college or job at some point, the need to constantly monitor the water resources is highly recommended.

**Table 6:** Carcinogenic risk assessment for Pb and Cr levels in the groundwater samples for dry and wet season.

	CR_ing_		
	Min	Mean	Max
Cr			
Adult (Dry season)	1.44E-03	1.48E-03	1.68E-03
Adult (wet season)	1.95E-04	1.45E-03	1.83E-03
Child (Dry season)	2.97E-03	3.45E-03	3.91E-03
Child (wet season)	4.56E-04	3.38E-03	3.83E-03
Pb			
Adult (Dry season)	3.81E-05	2.82E-03	5.51E-03
Adult (wet season)	2.02E-03	3.96E-03	9.61E-03
Child (Dry season)	7.84E-05	6.59E-03	1.29E-02
Child (wet season)	7.45E-03	9.45E-03	2.24E-02

Slope factor for Cr = 500 and Pb = 8.5 (μg /kg/day).

## Conclusion and recommendations

Groundwater is one of the sources of drinking water in Limpopo Province, South Africa. The quality of groundwater used in the Vybong region was investigated in this study using 17 selected boreholes. The findings show that the water quality seems appropriate for various domestic purposes except for drinking. This was due to contamination by *E. coli* and Total coliform in approximately 50% of the samples during the wet season. The anion concentrations, with the exception for nitrate, complied with the guidelines (SANS and WHO). The amount of Aluminium in all the boreholes exceeded the recommended threshold and therefore subject the residents to health risk.

Consequently, although the levels of trace metals recorded complied with the regulatory standards, the computation of the potential human health risk with the lifetime consumption of the water showed the possibility of both non-carcinogenic and carcinogenic risk especially in children. Thus, the potential dangers posed by the high presence of *E. coli*, total coliform, aluminium and nitrate necessitates relevant interventions such as point-of-use water treatment techniques in order to prevent any risks to the consumers due to microbial or chemical contamination.

## Data Availability

All the relevant data are included in this manuscript.
